# Ecological Impacts of Structural Racism on Health Disparity Through Its Determinants and Mediating Factors: A Case Study on Low Birthweight in Three Race/Ethnicity Groups in the United States

**DOI:** 10.3390/ijerph22050715

**Published:** 2025-05-01

**Authors:** Drona P. Rasali, Leanne L. Lefler, Chandra L. Ford, William D. Osei, Katharine T. Schaffzin

**Affiliations:** 1 Cecil C. Humphreys School of Law, University of Memphis, Memphis, TN 38103, USA; ktschffz@memphis.edu; 2 School of Population and Public Health, University of British Columbia, Vancouver, BC V6T 1Z3, Canada; 3 British Columbia Centre for Disease Control, Vancouver, BC V5Z 4R4, Canada; 4 Emotional Well Being Institute-Canada, Burnaby, BC V3N 1J2, Canada; wdosei@yahoo.ca; 5 Loewenberg College of Nursing, University of Memphis, Memphis, TN 38152, USA; lllefler@memphis.edu; 6 Department of African American Studies, Emory College of Arts and Science, Emory University, Atlanta, GA 30322, USA; chandra.l.ford@emory.edu; 7 Rollins School of Public Health, Emory University, Atlanta, GA 30322, USA

**Keywords:** structural racism, determinants of health disparity, low birthweight

## Abstract

Health disparities among populations across geographic regions, demographic and socio-economic groups are well documented; however, ecological studies which visually demonstrate health disparities associated with structural racism among racialized populations are limited. The purpose of this study was to examine low birthweight (LBW) as a measurable indicator of disproportionate health impacts across three race/ethnicity groups—non-Hispanic Black, Hispanic and non-Hispanic White–in the United States (US) for visualizing ecological manifestation of this disparity attributed to structural racism. We begin by providing the contextual background of structural racism through a literature review, and then more specifically, we examine LBW as a selected health indicator characterized with a socio-biological pathway of structural racism via socio-economic and politico–legal determinants and associated mediating factors to health disparities, from which we synthesized a visualization model with the indicators of structural racism reported in the literature reviewed. To further visualize these impacts, publicly available US County Health Ranking data for LBW, at the county level in two US states, Tennessee and Ohio, were analyzed to uncover area-based ecological health outcome—LBW. Significant correlation and scatter plots provided evidence of LBW as a racially sensitive health indicator associated with impacts of structural racism. These findings were further notable through examination of socio-economic determinants (e.g., race/ethnicity, income, education, and employment) and environmental factors such as housing issues as well as other underlying health conditions. Our case study has opened a window for visualizing disparity across non-Hispanic Black, Hispanic, non-Hispanic White populations as demonstrated by the prevalence of LBW disparity through its determinants and mediating factors at the county level. Potentially important policy implications for reparative change are drawn through our study findings that are salutary and/or reductive for addressing impacts of structural racism. Further studies are needed to fully understand the comprehensive web of area-based ecological factors impacting various health outcomes through the impacts of structural racism.

## 1. Background

Health inequities, the differences in health outcomes between populations, are avoidable and preventable, and are therefore unjust [1]. The evidence of extant disparities in health indicators, including morbidity and mortality among populations across geographic regions, demographic groups and socio-economic factors as determinants of health, has been officially reported in the United States (US) [2] and Canada [3,4] in the North American context. While both countries are grappling with extant structural racism, research on the various determinants associated with health disparities in Canada [5,6] and particularly in US life expectancy at the county level [7] among racial-ethnic groups has been reported. More specifically, several studies have reported racial discrimination against Black and African American people leading to health disparities that are structurally embedded in the US society [8,9,10,11,12]. Structural racism refers to the totality of ways in which societies embody racial discrimination through mutually reinforcing systems of housing, education, employment, earnings, benefits, credit, media, health care and criminal justice, while these patterns and practices in vogue at the system level, in turn, reinforce discriminatory beliefs, values and the distribution of resources [10]. A recent cross-disciplinary scoping review of the literature has comprehensively presented theoretical underpinnings and impacts of structural racism that are associated with health disparities across populations [13]. Yet, limited evidence visualizes ecological indicators of structural racism to health disparities among multiple racialized populations.

Birth outcomes such as low birthweight (LBW) serve as barometers of health system performance [14] and are generally reported as indicators of overall health and well-being of populations. Birth outcomes are significant risk factors for infant mortality, reflecting a serious public health concern that could have severe lifelong health impacts in terms of quality of life and costly societal burden [15,16]. In high-income North America, the US ranked the highest prevalence of LBW, 8.26% (95% UI, 8.15–8.36), as reported [14]. It is well documented in the US that racial disparities in birth outcomes are attributed to interpersonal and structural racism, but not due to other causes such as discrete genetic differences [15,17,18]; yet, the visualization of such disparities at the area-based ecological level has not been evidently demonstrated in the literature to inform the local-area-level policy planning for reparative changes.

An overwhelming body of empirical evidence shows that there exists an association of local-area (neighborhood) adversity factors such as poverty, segregation, crime, and eviction rates with poor infant outcomes [10,19]. Pervasive socio-economic differences occurring across race/ethnic subgroups are associated with resulting measures of disadvantage in birth outcomes, which continue from childhood to adulthood [20]. The physical, social, and political environments which are currently characterized through the contemporary social determinants of health are fundamentally rooted in structural racism [19]. For example, Black mothers are reported typically twice as likely to give birth to a LBW infant than their White counterparts; this Black–White disparity is due to impact of interpersonal or structural racism along with other risk factors such as mother’s age, education, smoking, and access to prenatal care [15,17,21].

One of the challenges of studying effects of structural racism on health is identifying appropriate geographical units for aggregation of the data to demonstrate its area-based impacts [16]. Thus, research to confirm the role of racism and to evaluate trends in the impact of racism on health outcomes by geographical areas has been hampered by the challenge of measuring racism [15]. In the empirical measurement of structural racism with its complex, insidious, and ecologic nature, residential segregation has been used as a key exposure factor and is highly predictive of a range of adverse birth outcomes [22], whereby three area-based measures of racial/ethnic composition serve as an indicator for residential segregation: the proportion of residents who are non-Hispanic Black, the proportion of residents of color, and the Index of Concentrations at the Extreme (ICE). The ICE measure captures geographical social polarization by looking at extremes of privilege and deprivation within a given neighborhood or census tract. In addition, exploitative revenue generation (the practice of using excessive municipal fines and fees) has a disproportionate impact on residents of color and is associated with preterm and LBW [22]. The discrete residential segregation in certain areas over time could potentially turn into area-based ecological diffusion of its impacts across a geographic jurisdiction. Therefore, we hypothesized that a measurable indicator can be identified at the county level for visualizing ecological impacts of structural racism in health outcomes such as LBW such that the ecological evidence can be used in local-level planning for reparative policy changes.

Thus, the purpose of this study is to examine and visualize the ecological distribution of low birthweight as an indicator of health disparity that shows evidence of structural racism across the populations of three race/ethnicity groups across the counties of Tennessee (TN) and Ohio (OH) states. Additionally, we examine some of the related multidimensional determinants and mediating factors at the county level within the two states.

## 2. Materials and Methods

The methodological framework of this study was developed simply to visualize how extant markers of structural racism identified in the literature actually manifest as area-based vivid realities for African American people compared to other racialized groups of non-Hispanic White people and Hispanic people in the US. We focus on LBW because it is considered sensitive to structural racism. As described below, we first reviewed the peer-reviewed literature to provide a contextual background for our study, then analyzed LBW rate data across counties in Ohio and Tennessee.

### 2.1. The Literature Review

We carried out a review of the peer-reviewed health science literature published from 2000 to 2023 that were indexed in PubMed^®^ online literature database hosted by the US National Library of Medicine, comprising more than 37 million citations for biomedical literature from MEDLINE, life science journals, and online books. The literature review was intended to contextualize the US background of structural racism as a determinant of health disparity measurable by low birthweight as a selected health indicator and the socio-biological pathways of structural racism associated with health disparities. Using three specific search terms, “Low birthweight”, “United States”, “Structural racism” in the PubMed platform, we found a total of 21 directly relevant peer-reviewed articles after removing duplicate or non-relevant entries. We extracted strikingly relevant excerpts from the full text of the selected articles and searched for relevant themes to identify socio-economic and politico–legal determinants, and mediating factors associated with low birthweight resulting from structural racism so as to guide the next steps in our study, i.e., data analysis and interpretation. Our review of the literature was intended to highlight the background context of the pervasive impact of structural racism systemically on adverse birth outcomes, particularly low birthweight among Black populations in the US.

### 2.2. Ecological Health Disparity Analysis of County-Level Data

Using publicly available US County Health Ranking data for the year 2021 extracted from the 2023 Annual Report of the Wisconsin University Population Health Initiative (URL Link for 2023 County Health Rankings National Findings Report—County Health Rankings & Roadmaps: https://www.countyhealthrankings.org/findings-and-insights/2023-county-health-rankings-national-findings-report, accessed on 17 November 2023), an ecological health disparity visualization analysis was carried out for area-based case studies of two selected states, one state in the deep South and another in the North, respectively, Tennessee and Ohio. The two states represent two different paths in the history of racial slavery in the US—Tennessee as a slave state deeply embedded in the Southern plantation system and Ohio as a free state with abolitionist influences. However, both states grappled with racial discrimination long after slavery was formally abolished. The two states provide appropriate representative samples for structural racism in the US. While the first author explored and conducted this research when stationed in Tennessee as a visiting scholar to the US, he also had the opportunity to visit Ohio, providing him a first-hand sense of the current geography and populations of both states during the time of this study in the fall 2023. The charts on the LBW data reported for all counties of the two states, measured in proportion of livebirths, were plotted against the proportions of non-Hispanic Black, Hispanic and non-Hispanic White populations by county to examine trends associated across the two variables. Correlation analyses using SPSS Version 25 software were conducted between LBW and the proportion of three racial-ethnic groups and selected other indicators that were hypothesized to have some link with the structural racism. Low birthweight is considered as a racism-sensitive health indicator, with its socio-economic and politico–legal policy inequities and mediating factors (e.g., race/ethnicity, income, education, and employment) and environmental factors (e.g., severe housing problems). We used the theoretical framework of structural racism with respect to measuring its area-based ecological impact on LWB across counties, whereas the area-based racial segregation as reviewed in the literature [22,23] would be localized within certain parts of the county. Thus, we were able to visualize disparity in the prevalence of LBW among non-Hispanic Black, Hispanic, non-Hispanic White populations across whole counties in the state. Finally, we have drawn potential policy implications for reparative change based on our findings for addressing the impacts of structural racism.

## 3. Results and Discussion

### 3.1. Literature Review for Contextual Background of Low Birthweight as Pathway for Structural Racism

We identified influencing determinants and mediating factors as having an effect on LBW and resulting from structural racism. We tabulated these factors into a summary table (Table 1). The included studies highlight the pervasive impact of structural racism on low birthweight and other adverse birth outcomes, particularly among Black populations in the US.

A total of 7 from the above-stated 21 selected US studies identified state- or county-level polices facilitating structural racism [15,17,18,19,22,29,33] that were negatively associated with LBW, while positive impact of policies were also reported [18,33] such as Safe Babes, Safe Mom and Paid Parent Leave supporting the birthing mothers. Other state- and county-level policy-related causes of structural racism negatively impacting birth weights reported in the literature include multiple level structural racism [21] and county-level eviction rates [36]. A Minnesota study showed that the higher risk of LBW for the US-born Black population compared to their White counterparts was explained by multidimensional structural racism of various typologies based on residential, income, education, employment, home ownership and criminal justice inequities [24,25], while inequities in three commonly known determinants of health—education, income and employment associated with structural racism resulting in LBW as birth outcome—were also reported [26]. Residential segregation typology of this association was also reported by other studies as well [23,36]. Criminal justice inequity was reported as a typology of structural racism associated with LBW by four studies [16,24,26,27]. Heteropatriarchal structural sexism were identified to explain structural racism associated with LBW in two studies [28,30]. The reported vulnerability factors of birthing mothers subject to impacts of structural racism on the birth outcome of LBW include their mental distress [27,34], life course impact such as Adverse Childhood Experiences (ACEs) and Adverse Adult Experiences (AAEs) [31]. The study that considered the status of prenatal mental health and substance use [34] indicated important implications for pregnant women as well as their developing children, and found positive associations between life course impact such as maternal childhood adversity (i.e., Adverse Childhood Experiences—ACEs) with prenatal mental health and substance use outcomes (e.g., severe anxiety, mood dysregulation measured by Mood Disorder Questionnaire—MDQ, and marijuana use) among urban, low-income, mostly minority women such as Black women. Further reports indicated the life course perspective with poorer birthing outcomes among Black people [31]. This originated from heightened exposure to stressors as adverse experiences early in life followed by cumulative exposure to the stressors later in life over time. Experiences of Discriminations (EoDs) at various settings such as school, job hiring, work, housing, medical care, sales and service, banking and mortgage, and in public settings and police or courts could impact on the psychophysiological status of pregnant women, leading to impairment of vasodilation during pregnancy [32]. This study found that impaired vasodilation during pregnancy as a potential mediating factor in the hypertension-related health disparities between African American women and European American women, and exposures to discrimination were associated with higher total peripheral resistance during pregnancy in the former with potential impact on the birth weight of their offspring [32].

While the report that Black mothers had dramatically higher rates of very low birthweight (VLBW) than White mothers [35], it was also reported that between 1989 and 2019 the relative odds that first births were VLBW increased by approximately 16 percent (from 0.030 to 0.034) for Black mothers and 13 percent (from 0.010 to 0.012) for White mothers. In addition, they showed that the maternal age-specific rate of VLBW had a widening gap with higher rates with the increasing age of the Black mothers compared to White mothers in 2017–2019. In summary, the most current literature provides the framework for using LBW as a lens for examining health disparities, such that LBW is characterized as a socio-biological pathway of structural racism. The framework necessitates visualization of these health disparities at the ecological level as vivid evidence of the impact of structural racism.

**Figure 1:**
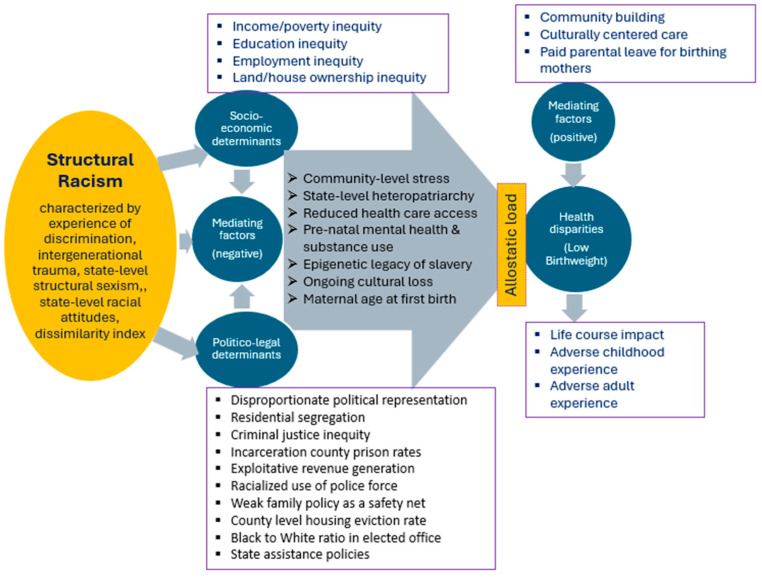
A model illustrating structural racism impacting socio-economic and politico–legal determinants, and mediating factors leading to low birthweight as a health disparity in racialized populations synthesized from the literature reviewed as presented in Table 1 (Shaded arrows indicate effects of the factors or their impacts on outcomes).

A pictorial chart model (Figure 1) was derived, summarizing the indicators of structural racism identified from the literature review, visualizing how it shows up in the characteristics of the population, how it impacts socio-economic and politico–legal determinants and mediating factors which, in turn, lead to health disparities such as low birthweight. The positive mediating factors are salutary to health equity, whereas negative ones are associated with health disparity. 

### 3.2. Ecological Case Study of Two US Counties

**Figure 2:**
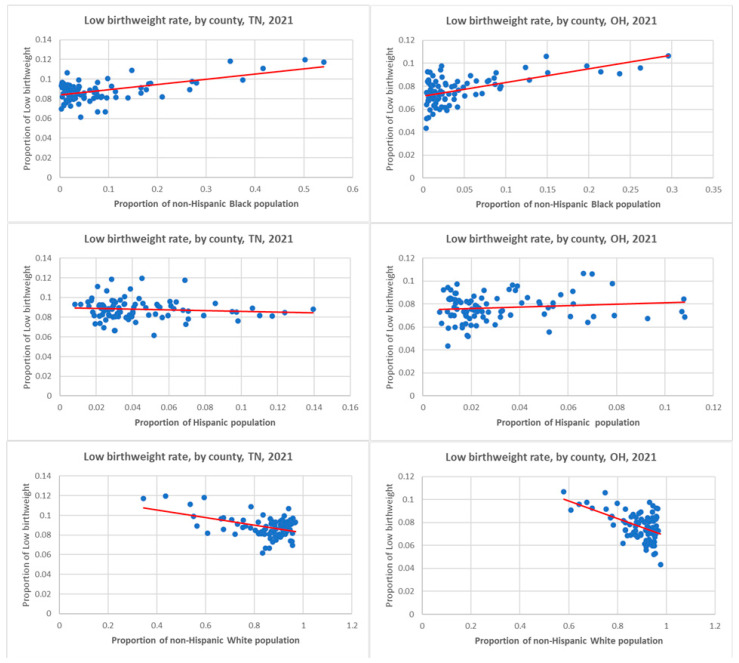
Scatter plots with each dot representing a county’s proportion and regression trend lines of low birthweight rate by the county’s proportion of non-Hispanic Black, Hispanic and non-Hispanic White populations in Tennessee (TN) and Ohio (OH) states.

County-level ecological socio-demographic and health characteristics of Tennessee and Ohio are summarized in Table 2. Tennessee in the US South, where slavery was legal, and Ohio in the US North, where slavery was illegal, are otherwise fairly comparable states in their demographic composition, though the former has a smaller population size. The former has also a relatively larger proportion of racialized populations, especially the non-Hispanic Black and has relatively lower status in health indicators such as birth outcomes, life expectancy, chronic disease especially diabetes that are generally reported to be linked with the impacts of structural racism. In particular among birth outcomes, LBW, can be considered as a case example indicator of health impact of structural racism in its local-area-based ecological analysis by counties, in both states showing certain distribution patterns as presented in scatter plots (Figure 2). In both states, the rate of LBW was lower in the counties that have lower proportion of non-Hispanic Black populations and increased with the proportion of that population within the county. Also in both states, the reverse pattern of lower LBW rate was observed with the increasing proportion of the non-Hispanic White population, while the Hispanic population did not show any significant pattern. These plots were consistent with Pearson’s correlations of LBW rate, which were significantly positive with the proportion of the non-Hispanic Black population (*p* < 0.01) and significantly negative with the proportion of the non-Hispanic White population (*p* < 0.01), whereas the Hispanic population was statistically non-significant in both states. It must be noted that the non-Hispanic Black population had suffered from extreme racial discrimination with the historical enslavement, followed by a series of racially discriminatory policies as illustrated in Figure 1, and their health outcomes such as LBW had been severely impacted by the socio-economic and politico–legal policy determinants and associated mediating factors. On the other hand, the Hispanic population might not have been subjected to racial discrimination practices that were of the same type or severity as the former. We suggest that this would help explain the difference in the health outcomes between the two populations.

Using Pearson’s correlation analysis, further exploration of selected measurable indicators that have been generally considered examples of mediating factors associated with the impacts of structural racism (Table 2) showed that Education (Highschool completion in TN, Highschool graduation in OH, school segregation), Income (Income inequality, Median household income, Childhood poverty, Unemployment, Food insecurity, and Health (Life expectancy, Frequent physical stress, Diabetes prevalence, HIV prevalence) were all significantly correlated towards disadvantageousness for LBW. The selection of these measurable indicators for correlation analysis as presented in Table 3 was an opportunistic exercise, as the indicators were intuitively selected based on their relevance to the findings of the literature review with respect to impact of structural racism on the selected health outcome, LBW, from the available indicators in the database used. 

Our case study-based analysis of LBW provides visualized ecological evidence of health disparities, particularly between non-Hispanic White and non-Hispanic Black populations to the disadvantage of the latter. We were able to show the nature of health disparity structurally embedded in the influencing factors measurable at the county level as elucidated by the racially disproportionate ecological distribution of LBW. Particularly, our two-state case study supports the notion that residential segregation is a reflection and reinforcement of structural and institutional racism resulting from racialized and economically segregated neighborhoods [10,23,24].

Mehra et al. introduced five distinct modes of operationalizations of segregation that could explain its impact at the county level [23]. These five modes that could be calculated for indexing cross micro-level spatial units (e.g., neighborhoods) within macro-level spatial units (e.g., regions) are comprised of Exposure—the degree of neighborhood isolation or interaction of minority with majority groups; Evenness—the degree to which each neighborhood has the same proportion of minority and majority members for even distribution; Clustering—the degree to which minority neighborhoods are contiguous and tightly clustered; Concentration—the degree to which minority members occupy a small proportion of the total area of a region; and Centralization—the degree to which a minority group is centrally located within a region. These modes of segregation could explain how the proportions of the LBW rate are positively and significantly correlated with the proportion of the non-Hispanic Black population and negatively correlated with the non-Hispanic White population, in both states studied. However, interestingly in our case studies, the Hispanic population did not show these patterns of significant health disparity at the ecological level, even though it could have been subjected to county-level exploitative revenue generation through fees and fines as described by Davies et al. [22]. Yet, it is important to note that Hispanics have not previously been reported to have these five modes of segregation, and the ethnic/racial discrimination against them was not the same in terms of its form and severity as in the non-Hispanic Black population to cause similar impact at the ecological level, and this may be an area for future study.

The disproportionate representation of Black people in the US penal system demonstrates longstanding mechanisms underlying inequities in incarceration (dating back to colonialism) and health at the population level [10]. The ecological concentration of incarceration measured as county-level prison rates has been reported to be associated with racial disparities in adverse birth outcome such as LBW mediated through two pathways—community-level mental stress and reduced health care access [27].

Inequity in education has been identified in the literature [24,26], and constitutes one determinant of the multidimensional structural racism typology which could be a factor explaining birth inequities leading to a higher risk of LBW for the US-born Black population [24]. This further resonates with our county-level results showing significant correlations of LBW with a degree of High School completion in Tennessee, High School graduation in Ohio and a degree of school segregation in both states as valid indicators of education (Table 2). Likewise, our background literature review indicated income, poverty and employment as three other determinants of the multidimensional structural racism typology, explaining the higher risk of LBW among the Black population [24,25,26,29]. These findings were consistent with significantly positive correlations of LWB with income inequality (negative correlation with median household income), child poverty and unemployment at the county level in both the states (Table 2).

Closely related with the residential segregation, homeownership has been well documented as one of the major components embedded in the structural racism in the US leading to health disparity such as impacting negatively on birth outcomes in the Black population [9,19,25,26]. This pattern of relationships has been consistently elucidated by the significant correlations of LBW negatively with homeownership and positively with severe housing problems at the county level in both the states.

Some of the factors intrinsic to the birthing Black mothers reported in the literature influencing their disproportionate rate of LWB include life course impacts of Adverse Childhood Experiences (ACEs) and Adverse Adult Experiences [31,34], prenatal mental health and substance use [34], maternal age at first births [35], as well as the epigenetic legacy of enslavement and ongoing cultural loss [21], which are all factors that are structural in nature. Furthermore, significant correlations of LBW found with other selected health status indicators, such as being negatively correlated with life expectancy, and positively with frequent physical distress, HIV prevalence and food insecurity at the county level in both states could indicate their respective association embedded as factors mediating structural racism, similar to those of community-level mental stress and reduced health care access reported [27].

*Allostatic load.* In the recent scoping review, it was surmised [13] that Black Americans suffered from chronic stresses on an ongoing basis, bearing a higher allostatic load as compared to White individuals. The concept of allostatic load, first introduced by McEwen and Stellar [37], is the physio-pathological impact of wear and tear resulting from chronic stresses on a number of organs and tissues that can predispose the organism to disease. It is crucial in understanding health inequalities, particularly in the context of our study on LBW among non-Hispanic Black women compared to their non-Hispanic White counterparts. It refers to the physiological cost of chronic exposure to fluctuating or heightened neural and neuroendocrine responses due to repeated or chronic environmental stressors [38]. Ethnicity has been associated with varying levels of allostatic load, with Black Americans generally exhibiting higher levels than the White population [38], while everyday racial discrimination, as well as institution-specific structural discrimination, differentially affect allostatic load among Black women, with higher levels reported in those experiencing greater perceived racial or social adversities throughout their lives [38]. While general population studies consistently show that low socio-economic status, impoverished neighborhoods, and low educational attainment increase allostatic load through the mechanism of neuronal and hormonal responses to the chronic stresses caused by those factors, the evidence of specific impact of allostatic load on birth outcomes is limited, warranting further investigation to establish a clear understanding of the associated multidimensional factors.

Finally, state- or county-level policies, as reported in our literature review, that have a salutary impact against structural racism including aid to needy families, housing assistance, Medicaid, minimum wage, earned income tax credits [17], political representation of racialized minorities [19], the Safe Babies, Safe Moms initiative [18], paid parental leave [33], community building as well as culturally centered care [21] could have reparative impacts on health disparity measured by the indicator such as LBW. On the other hand, implementation of these policies has the potential to not only positively impact but at the same time reinforce structural racism, such as the case of racialized police use of force [16], reductive racial bias by the state [15], exploitative revenue generating county-level fees and fines (Davis et al., 2023), county-level housing eviction rates [36]. As such, policy implementation could have reductive association leading to a higher LBW rate in counties with a higher proportion of racialized populations.

Some of the limitations of our study include the list of variables included in the correlations analysis of LBW was not exhaustive enough to draw the comprehensive web of influencing mechanisms through the pathways of socio-economic and politico–legal policy determinants and mediating factors of structural racism impacting on racial health disparity in LBW. Our analyses were intended to simply examine individual association of the variables without exploring their interdependence. A robust analysis of desirable data involving statistical rigor such as the path coefficient or structural equation modeling, which is out of the scope for our study, would be a future aspirational exercise for deriving comprehensive results on the mechanisms of the whole system of structural racism. In summary, structural racism creates a web of interrelated factors including socio-economic and circumstantial inequities, reduced health care access, residential segregation, chronic stress, and biological mechanisms that collectively contribute to the higher incidence of LBW among non-Hispanic Black mothers. Addressing the multifactorial issues of structural racism requires comprehensive reparative policy changes for interventions aimed at reducing racial disparities and promoting health equity. Further studies are needed to fully understand the comprehensive web of area-based ecological factors affecting various health outcomes through the impacts of structural racism.

## 4. Conclusions

In the backdrop of the pictorial model (Figure 1) derived from our literature review and visualization of ecological scatter plot charts (Figure 2) from our data analysis, we found that while some determinants such as higher rates of high school completion/graduation, median household income, homeownership, and life expectancy are positively (i.e., favorably) correlated with lower LBW rates, other determinants such as school segregation, income inequality, child poverty, unemployment, severe housing problems, frequent physical distress, diseases (e.g., diabetes and HIV), and food insecurity emerge as significant deterrents to healthier LBW outcomes. The consistency of these associations across counties in both US states (Table 3) underscores their embodiment of structural racism. This provides visualization of the ecological impacts of structural racism on LBW disparities, particularly among non-Hispanic Black populations, compared to their non-Hispanic White counterparts, presenting compelling evidence of the area-level impacts of structural racism.

To address the multifactorial nature of structural racism including discrimination embedded in labor markets, residential segregation, income inequality, and challenges to fair political representation, we require multifaceted policy interventions, community-level changes, and systemic reforms targeting the various dimensions of structural racism. Effective solutions must also consider the historical context and cumulative stressors affecting marginalized communities through intergenerational trauma leading to allostatic load. The background literature review and ecological analysis of LBW in our two-state case study generally corroborated each other: extant health disparities between non-Hispanic White and non-Hispanic Black populations are associated with area-level structural racism-related socio-economic determinants and mediating factors. The racially disproportionate county-level distribution of LBW along with various influencing determinants and mediating factors elucidate the nature of health disparity structurally embedded in US society. The racially disproportionate ecological concentration of the rates of residential segregation, incarceration, inequities in education, income and employment, some measurable birthing mother traits, and racially biased policies at the state or county level constitute the components of structural racism that lead to disparity in health such as LBW and need policy change considerations. Future research should further examine the intersectional (i.e., interlocking) relationships by which structural racism and other structural forces (e.g., sexism) jointly exacerbate reproductive health inequities [39].

## Figures and Tables

**Table 1:** Summary of included US studies with indicators of structural racism associated with low birthweight.

Author(s) (Year of Publication)	Indicator(s) of Structural Racism as Mediating Factors of Impact on Low Birthweight	Extract from the Relevant Findings
Chantarat et al. (2022a) [24]	Residential segregation Education inequity Employment inequity Income inequity Homeownership inequity Criminal justice inequity	Structural racism, measured across dimensions such as residential, income, education, employment, home ownership, and criminal justice, significantly predicts higher LBW rates among US-born Black Minnesotans compared to the White population, while the LBW incidence for Black infants was more than double that of White infants. The study implied that multisectoral policy solutions are necessary to dismantle structural racism and reduce birth inequities
Chantarat et al. (2022b) [25]	Employment—labor markets measured by commuting zones	Structural racism in labor markets adversely affects US-born Black pregnant people in the South, leading to higher LBW rates. This study implied that regional differences in labor market racism must be addressed to mitigate LBW disparities
Wallace et al. (2015) [26]	Education inequity Income inequity/inequality Employment Incarceration	High levels of racial and socio-economic inequality increase the risk of Small-for-Gestational-Age (SGA) births, particularly for Black populations. The study indicated that addressing both racial and income inequalities is crucial for improving birth outcomes
Sonderlund et al. (2023) [27]	● Incarceration—county-level prison rates ● Mediated by: ○ (1) Community-level mental distress ○ (2) Reduced health care access	Community mental distress and health care access mediate the relationship between mass incarceration and adverse birth outcomes. This study implied that interventions should target mental health and health care access to mitigate the effects of structural racism
Everett et al. (2022) [28]	Structural heteropatriarchy—state-level LGB-policies, family planning policies, and indicators of structural sexism (e.g., women’s political and economic position relative to men)	Higher levels of heteropatriarchy were linked to increased risk of lower birthweight, though not significantly for LBW clinical cutoffs, indicating also that further research is needed to explore the impacts of heteropatriarchy on birth outcomes
Chambers et al. (2018) [29]	● County-level structural racism indicators: ○ Traditional (e.g., dissimilarity index) ○ Novel indicators (e.g., Black to White ratio in elected office) ○ County-level poverty and racial disparities in incarceration included separately and jointly	County-level indicators of structural racism, including poverty and racial disparities in incarceration, are significantly associated with birth weight, suggesting that innovative measures of structural racism are needed at the county level, along with policy reforms
Mehra et al. (2017) [23]	Racial residential segregation	Segregation is one of the key structural determinants of racial inequality that contribute to adverse birth outcomes, including LBW, and suggested that policies addressing residential segregation are crucial for improving birth outcomes
Chegwin et al. (2023) [16]	Racialized police use of force (PUOF)	Racialized police violence is associated with higher LBW rates among Black women, suggesting that addressing police violence is necessary to improve health outcomes for Black communities
Nagle and Samari (2019) [30]	State-level structural sexism	Studying state-level structural sexism and cesarean sections in the US did not have significant findings related to LBW, suggesting further research needed to explore structural sexism’s impact on birth outcomes
Pearlman and Robinson (2022) [17]	Five state-level policies: Aid to Families with Dependent Children/Temporary Aid for Needy Families, housing assistance, Medicaid, minimum wage, and the earned income tax credit (EITC) as safety net for risk reduction in LBW	Black mothers face higher LBW risks due to structural racism, with policy interventions like earned income tax credit (EITC) and Aid to Families with Dependent Children/Temporary Aid for Needy Families (AFDC/TANF) potentially mitigating these disparities, and recommended policy reforms targeting economic support to reduce LBW disparities
Nguyen et al. (2020) [15]	State-level racial attitudes (Twitter derived)	Negative racial sentiments correlate with adverse birth outcomes for minorities, and recommended promotion of respect and inclusion that can improve birth outcomes
Patchen et al. (2023) [18]	Safe Babies, Safe Moms—initiative (positive for trauma-informed supports)	Trauma-informed health care models are essential for addressing the adverse impacts of structural racism, and suggested that health care systems need to adopt trauma-informed approaches
Mersky et al. (2023) [31]	Life course impact Adverse Childhood Experiences (ACEs) and Adverse Adult Experiences (AAEs)	Studying association of adverse experiences with pregnancy and birth outcomes showed that cumulative stress from racism contributes to poor birth outcomes for Black women, suggesting that systemic changes are needed to address these chronic stressors
Stanhope et al. (2023) [19]	Political representation—country-level racial representation	Political representation disparities at the county level in Georgia are associated with LBW disparities, suggesting increasing political representation for marginalized groups in order to improve their birth outcomes
Bridgeman-Bunyoli et al. (2022) [21]	● Three intersecting and predisposing root causes: ○ Systemic racism at multiple levels; ○ The epigenetic legacy of enslavement; and ○ Ongoing cultural loss. Two additional themes for healing: ○ (4) Community building; and ○ (5) Culturally centered care.	The study explained how historical racism and associated chronic stress could lead to high LBW rates among Black women, implying that addressing historical and ongoing racism is critical for improving birth outcomes
Christian et al. (2021) [32]	Experiences of discriminations (EODs) leading to impairment of vasodilation during pregnancy: (1) at school, (2) getting hired or getting a job, (3) at work, (4) getting housing, (5) getting medical care, (6) getting service in a store or restaurant, (7) getting credit, bank loans or a mortgage, (8) on the street or in a public setting, and (9) from the police or in the courts. Additionally, two measures as responses to poor treatment with high and low scores.	Vascular dysfunction linked to racism contributes to lower birth weights among African Americans, and suggested that addressing vascular health disparities can improve birth outcomes
Davis et al. (2023) [22]	Exploitative revenue generation—county-level fees and fines	County-level fees that are exploitative revenue sources are associated with higher LBW rates, especially among Black and Latino mothers, and suggested that reforming exploitative financial policies can reduce LBW disparities
Karasek et a. (2022) [33]	Paid Parental Leave Ordinance (PPLO) as support to birthing mothers	No significant association was evident between paid parental leave and birth outcomes; however, further research is needed to understand the effects of paid leave on birth outcomes
Jasthi et al. (2022) [34]	Adverse Childhood Experiences (ACEs); Prenatal Mental Health and Substance Use	Adverse childhood experiences and poor mental health during pregnancy are linked to LBW, suggesting that addressing childhood adversity and prenatal mental health can improve birth outcomes
Geronimus et al. (2023) [35]	Maternal age at first births; Weak Family policies as safety net	Increased very low birthweight (VLBW) rates among Black mothers are linked to socio-economic factors and maternal age, implying that targeted interventions for older Black mothers can reduce VLBW disparities
Harville et al. (2022) [36]	Access to housing: County-level eviction rates	Housing eviction is a social determinant of pregnancy health such that high eviction rates are associated with increased LBW rates, particularly among Black mothers, and recommended policies addressing housing stability as essential for improving birth outcomes

**Table 2:** Socio-demographic and health characteristics of Tennessee and Ohio, by county, 2021.

Socio-Demographic and Health Data	US States	
	Ohio (n = 88)	Tennessee (n = 95)
State Total Population	11,780,017	6,975,218
% non-Hispanic Black	12.8	16.6
% Hispanic	4.3	6.1
% Asian	2.76	2.0
% Non-Hispanic White	77.7	73.1
Poor or Fair Health, %	14.5	15.7
Low Birthweight, % births	8.6	9.1
Teen Births, per 1000 live births	20.9	27.2
Life Expectancy, years	76.5	75.3
Diabetes Prevalence, %	10.9	12.5
Air Pollution Particulate Matter (PM2.5)	8.9	7.6
Severe Housing Problems, %	13.1	13.4

n = the number of counties.

**Table 3:** Pearson’s correlations of low birthweight rate with three race/ethnicity groups and selected measures of structural racism across county-level data points in Tennessee and Ohio, 2021.

	Tennessee		Ohio	
	R	(n)	R	(n)
Non-Hispanic Black	0.557 **	95	0.584 **	88
Hispanic	−0.102	95	0.127	88
Non-Hispanic White	−0.459 **	95	−0.512 **	88
High School Completion	−0.202 *	95	0.116	88
High School Graduation	−0.203	90	−0.401 **	88
School Segregation	0.249 *	90	0.485 **	87
Income Inequality	0.263 *	95	0.594 **	88
Median Household Income	−0.294 **	95	−0.391 **	88
Children in Poverty	0.379 **	95	0.600 **	88
Unemployment	0.559 **	95	0.539 **	88
Homeownership	−0.404 **	95	−0.548 **	88
Severe Housing Problems	0.473 **	95	0.526 **	88
Life Expectancy	−0.348 **	95	−0.549 **	88
Frequent Physical Distress	0.329 **	95	0.260 *	88
Diabetes Prevalence	0.620 **	95	0.529 **	88
HIV Prevalence	0.398 **	91	0.528 **	86
Food Insecurity	0.339 **	95	0.528 **	88

R = Pearson’s correlation co-efficient with a ** highly significant *p*-value at <0.01 and a * significant *p*-value at <0.05; n = number of county data points within the state.

## Data Availability

1. We reviewed peer-reviewed articles found in our search at PubMed. 2. We used publicly available US County Health Ranking data for the year 2021 extracted from the 2023 Annual Report of the Wisconsin University Population Health Initiative (URL Link for 2023 County Health Rankings National Findings Report—County Health Rankings & Roadmaps: (https://www.countyhealthrankings.org/findings-and-insights/2023-county-health-rankings-na-tional-findings-report, accessed on 17 November 2023).

## References

[1] Whitehead M. (1992). The Concepts and Principles of Equity and Health. Int. J. Health Serv..

[2] Centers for Disease Control and Prevention (US CDC) (2011). CDC Health Disparities and Inequalities Report United States.

[3] Public Health Agency of Canada (PHAC) (2018). Key Health Inequalities in Canada: A National Portrait.

[4] Rasali D., Kao D., Fong D., Qiyam L. (2019). Priority Health Equity Indicators for British Columbia: Preventable and Treatable Premature Mortality.

[5] Rasali D., Li C., Mak S., Rose C., Janjua N., Patrick D. (2021). Correlations of COVID-19 Incidence with Neighborhood Demographic Factors in BC. Ann. Epidemiol..

[6] Brown T.H., Homan P.A. (2022). Frontiers in Measuring Structural Rcism and its Health Effects. Health Serv. Res..

[7] GBD US Health Disparities Collaborators (2022). Life Expectancy by County, Race, and Ethnicity in the USA, 2000 19: A Systematic Analysis of Health Disparities. Lancet.

[8] Gee G.C., Ford C.L. (2011). Structural Racism and Health Inequities: Old Issues, New Directions. Du. Bois Rev. Social. Sci. Res. Race.

[9] Krieger N. (2014). Discrimination and Health Inequities. Int. J. Health Serv..

[10] Bailey Z.D., Krieger N., Agénor M., Graves J., Linos N., Bassett M.T. (2017). Structural Racism and Health Inequities in the USA: Evidence and Interventions. Lancet.

[11] Sweeting J.A., Akinyemi A.A., Holman E.A. (2023). Parental Preconception Adversity and Offspring Health in African Americans: A Systematic Review of Intergenerational Studies. Trauma Violence Abus..

[12] Williams D.R., Lawrence J.A., Davis B.A. (2019). Racism and Health: Evidence and Needed Research. Annu. Rev. Public Health.

[13] Rasali D.P., Woodruff B.M., Alzyoud F.A., Kiel D., Schaffzin K.T., Osei W.D., Ford C.L., Johnson S. (2024). Cross-Disciplinary Rapid Scoping Review of Structural Racial and Caste Discrimination Associated with Population Health Disparities in the 21st Century. Societies.

[14] Global Nutrition Target Collaborators (2025). Global, Regional, and National Progress towards the 2030 Global Nutrition Targets and Forecasts to 2050: A Systematic Analysis for the Global Burden of Disease Study 2021. Lancet.

[15] Nguyen T.T., Adams N., Huang D., Glymour M.M., Allen A.M., Nguyen Q.C. (2020). The Association Between State-Level Racial Attitudes Assessed from Twitter Data and Adverse Birth Outcomes: Observational Study. JMIR Public Health Surveill..

[16] Chegwin V., Teitler J., Muchomba F.M., Reichman N.E. (2023). Racialized Police Use of Force and Birth Outcomes. Social Sci. Med..

[17] Pearlman J., Robinson D.E. (2022). State Policies, Racial Disparities, and Income Support: A Way to Address Infant Outcomes and the Persistent Black-White Gap?. J. Health Politics Policy Law..

[18] Patchen L., McCullers A., Beach C., Browning M., Porter S., Danielson A., Asegieme E., Richardson S.R., Jost A., Jensen C.S. (2024). Safe Babies, Safe Moms: A Multifaceted, Trauma Informed Care Initiative. Matern. Child Health J..

[19] Stanhope K.K., Kapila P., Umerani A., Hossain A., Salah M.A., Singisetti V., Carter S., Boulet S.I. (2023). Political Representation and Perinatal Outcomes to Black, White, and Hispanic People in Georgia: A Cross-sectional Study. Ann. Epid..

[20] Blumenshine P., Egerter S., Barclay C.J., Cubbin C., Braveman P.A. (2010). Socioeconomic Disparities in Adverse Birth Outcomes: A Systematic Review. Am. J. Prev. Med..

[21] Bridgeman-Bunyoli A.M., Cheyney M., Monroe S.M., Wiggins N., Vedam S. (2022). Preterm and Low Birthweight Birth in the United States: Black Midwives Speak of Causality, Prevention, and Healing. Birth,.

[22] Davis B.A., Arcaya M.C., Williams D.R., Krieger N. (2023). The impact of County-level Fees & Fines as Exploitative Revenue Generation on US Birth Outcomes 2011–2015. Health Place.

[23] Mehra R., Boyd L.M., Ickovics J.R. (2017). Racial Residential Segregation and Adverse Birth Outcomes: A Systematic Review and Meta-analysis. Soc. Sci. Med..

[24] Chantarat T., Van Riper D.C., Hardeman R.R. (2022). Multidimensional Structural Racism Predicts Birth Outcomes for Black and White Minnesotans. Health Serv. Res..

[25] Chantarat T., Mentzer K.M., Van Riper D.C., Hardeman R.R. (2022). Where are the Labor Markets?: Examining the Association between Structural Racism in Labor Markets and Infant Birth Weight. Health Place.

[26] Wallace M.E., Mendola P., Liu D., Grantz K.L. (2015). Joint Effects of Structural Racism and Income Inequality on Small-for-Gestational-Age Birth. Am. J. Public Health.

[27] Sonderlund A.L., Williams N.L., Charifson M., Ortiz R., Sealy-Jefferson S., De Leon E., Schoenthaler A. (2023). Structural Racism and Health: Assessing the Mediating Role of Community Mental Distress and Health Care Access in the Association between Mass Incarceration and Adverse Birth Outcomes. SSM—Popul. Health.

[28] Everett B.G., Limburg A., Homan P., Philbin M.M. (2022). Structural Heteropatriarchy and Birth Outcomes in the United States. Demography.

[29] Chambers B.D., Erausquin J.T., Tanner A.E., Nichols T.R., Brown-Jeffy S. (2018). Testing the Association Between Traditional and Novel Indicators of County-Level Structural Racism and Birth Outcomes among Black and White Women. J. Racial Ethn. Health Disparities.

[30] Nagle A., Samari G. (2021). State-level Structural Sexism and Cesarean Sections in the United States. Soc. Sci..

[31] Mersky J.P., Jeffers N.K., Lee C.P., Shlafer R.J., Jackson D.B., Gómez A. (2024). Linking Adverse Experiences to Pregnancy and Birth Outcomes: A Life Course Analysis of Racial and Ethnic Disparities Among Low—Income Women. J. Racial Ethn. Health Disparities.

[32] Christian L.M., Koenig J., Williams D.P., Kapuku G., Thayer J.F. (2012). Impaired Vasodilation in Pregnant African Americans: Preliminary Evidence of Potential Antecedents and Consequences. Psychophysiology.

[33] Karasek D., Raifman S., Dow W.H., Hamad R., Goodman J.M. (2022). Evaluating the Effect of San Francisco’s Paid Parental Leave Ordinance on Birth Outcomes. Int. J. Environ. Res. Public Health.

[34] Jasthi D.L., Nagle-Yang S., Frank S., Masotya M., Huth-Bocks A. (2012). Associations Between Adverse Childhood Experiences and Prenatal Mental Health and Substance Use Among Urban, Low—Income Women. Community Ment. Health J..

[35] Geronimus A.T., Bound J., Hughes L. (2023). Trend Toward Older Maternal Age Contributed to Growing Racial Inequity in Very-Low-Birthweight Infants in the US. Health Aff..

[36] Harville E.W., Wallace M.E., Theall K.P. (2022). Eviction as a Social Determinant of Pregnancy Health: County-level Eviction Rates and Adverse Birth Outcomes in the United States. Wiley-Health Soc. Care Community.

[37] McEwen B.S., Stellar E. (1993). Stress and the Individual. Mechanisms Leading to Disease. Arch. Intern. Med..

[38] Guidi J., Lucente M., Sonino N., Fava G.A. (2021). Allostatic Load and Its Impact on Health: A Systematic Review. Psychother. Psychosom..

[39] Alson J.G., Robinson W.R., Pittman L., Doll K.M. (2021). Incorporating Measures of Structural Racism into Population Studies of Reproductive Health in the United States: A Narrative Review. Health Equity..

